# Supplementary material for: DSCAM-AS1-Driven Proliferation of Breast Cancer Cells Involves Regulation of Alternative Exon Splicing and 3′-End Usage
Source: Cancers (Basel). 2020 Jun 3;12(6):1453. doi: 10.3390/cancers12061453 (PMC7352480; doi:10.3390/cancers12061453)
Supplement: Supplementary file 1 [file cancers-12-01453-s001.zip › cancers-782617-supplementary/cancers-782617-supplementary-figures.pdf]

cancers

MDPI

Article

DSCAM-AS1-Driven Proliferation of Breast Cancer Cells Involves Regulation of Alternative Exon Splicing and 3′-End Usage

Jamal Elhasnaoui, Valentina Miano, Giulio Ferrero, Elena Doria, Antonette E. Leon, Aline S.C. Fabricio, Laura Annaratone, Isabella Castellano, Anna Sapino and Michele De Bortoli

Supplementary Figures

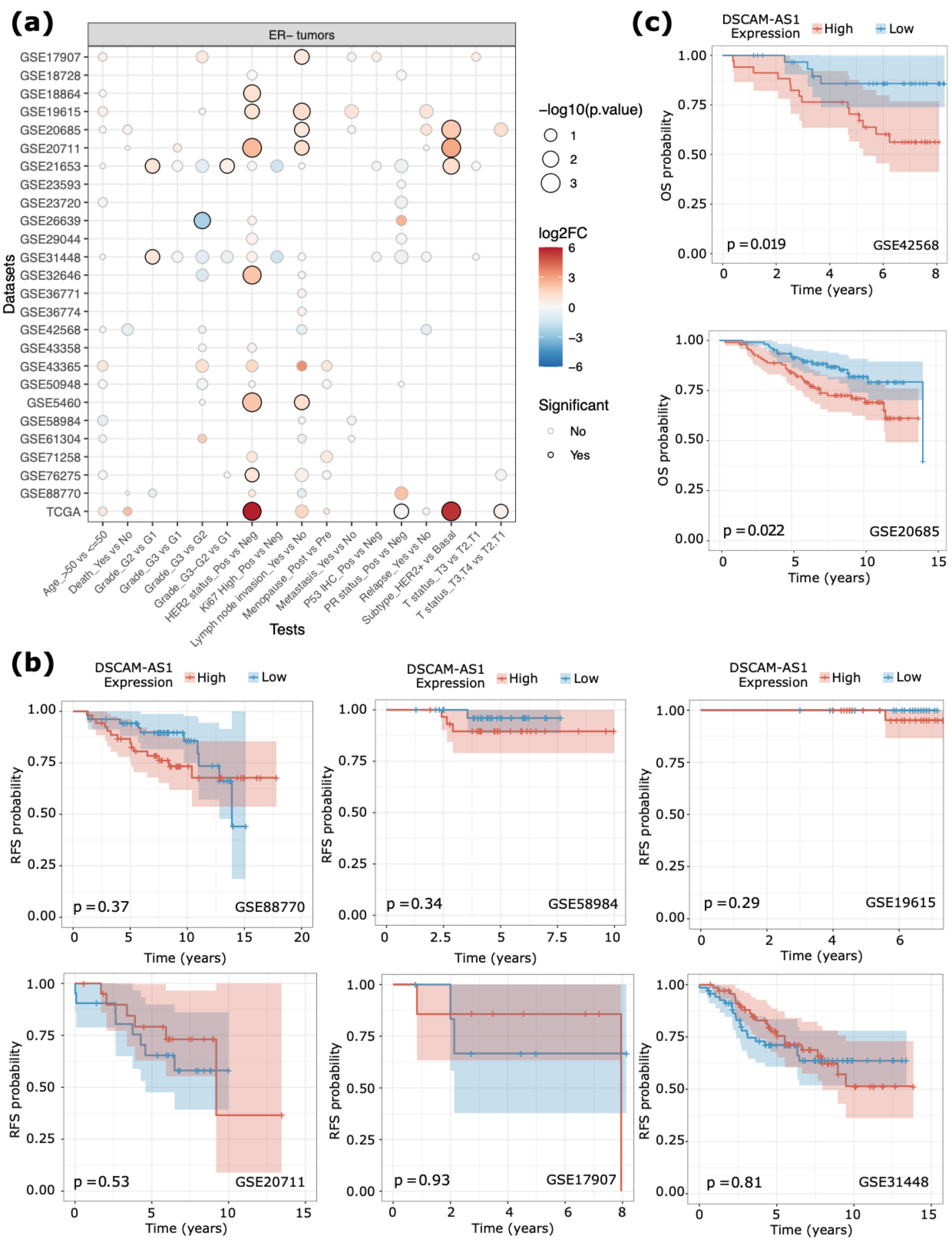

Figure S1. (**a**) Dot plot reporting the level of statistical significance of the differential *DSCAM-AS1* expression analyses between groups of ER-negative BC patients separated with respect to specific

clinical data. The size of the dot is proportional to the significance of the results while the color code represents the log2FC of expression. ER, Estrogen Receptor; Pos, positive; Neg, negative; PR, Progesterone Receptor. (**b**) Kaplan-Meier curves representing the datasets with nonsignificant difference in Relapse Free Survival (RFS) of BC patients based on the median level of DSCAM-AS1 expression. *p*-value by log-rank test. (**c**) Kaplan-Meier curves representing the Overall Survival (OS) of BC patients based on the median level of DSCAM-AS1 expression. *p*-value by log-rank test.

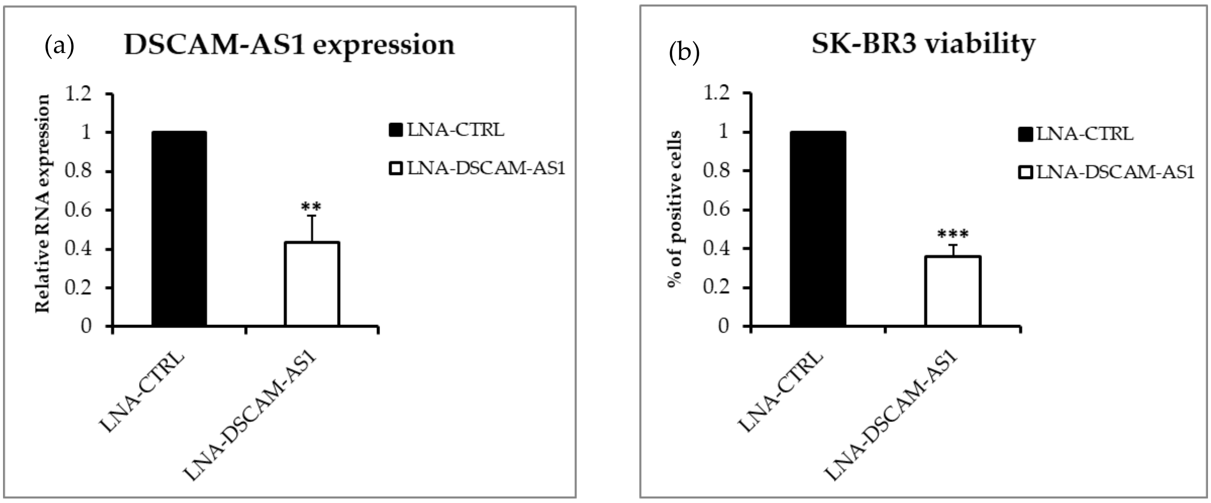

Figure S2. (**a**) Expression levels of *DSCAM-AS1* and (**b**) viability measure by Crystal Violet Assay in SK-BR-3 cells upon transection of *DSCAM-AS1*-targeting or control LNA. Error bars represent the standard deviation of three biological replicates. Significance from T-test: **, p-value < 0.01; ***, p-value < 0.001.

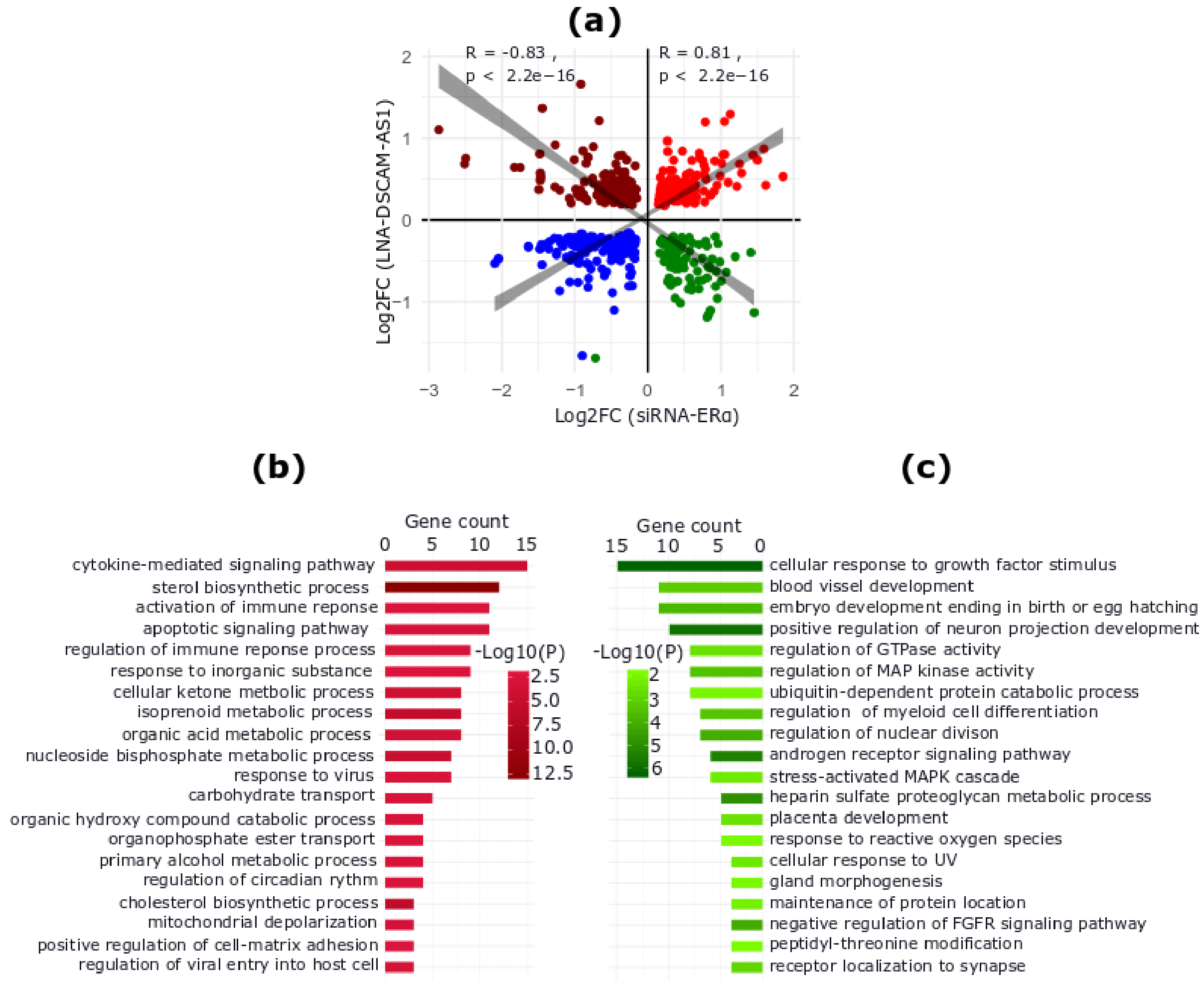

Figure S3. (**a**) The scatter plot shows the log2 fold change of DE genes between this study and those obtained upon ERα silencing (siERα) [13]. Dark red and green dots represent genes upregulated in this study while downregulated in the siERα experiment, and those downregulated in this study and upregulated in the siERα experiment, respectively. Blue and red dots represent those genes downregulated or upregulated in both studies, respectively. (**b**) GO terms enriched for genes upregulated in this study and downregulated in the siERα experiment. (**c**) GO terms enriched for genes downregulated in this study while upregulated in the siERα experiment.

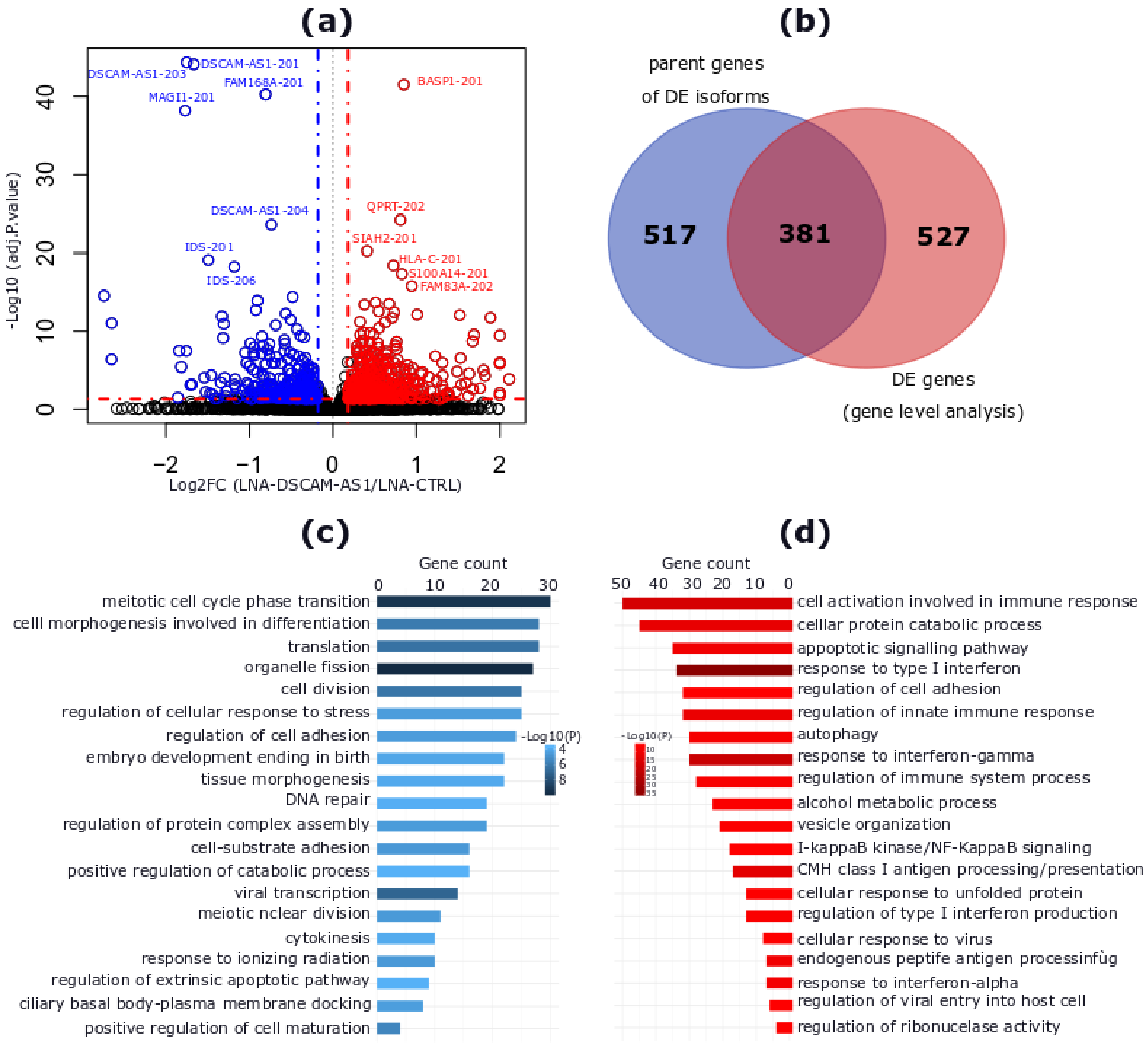

Figure S4. (**a**) Volcano plot showing the log2FC of gene expression and the statistical significance of the differential expression (DE) analysis at isoform level performed between MCF-7 cells transfected with control or *DSCAM-AS1*-targeting LNA GapmeRs. In red are reported the up-regulated isoforms while in blue the down-regulated ones. (**b**) Venn diagram showing the overlap between DE genes and parent genes of DE isoforms. (**c–d**) Enriched GO biological processes related to parent genes of downregulated and upregulated isoforms, respectively.

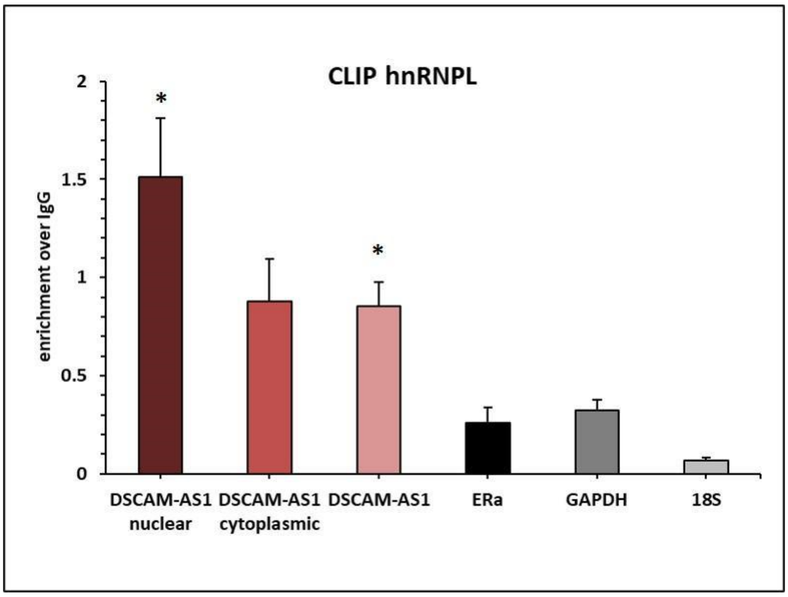

Figure S5. Cross-linking ImmunoPrecipitation (CLIP) of hnRNPL in MCF-7 cells shows evidence for physical interaction with *DSCAM-AS1* transcripts, considering separately the nuclear and the cytoplasmic isoforms. ERα, GAPDH and 18S were used as the negative control. Error bars represent the standard error of three biological replicates. Significance from T-test (*DSCAM-AS1* versus 18S enrichment): *, p-value < 0.05.

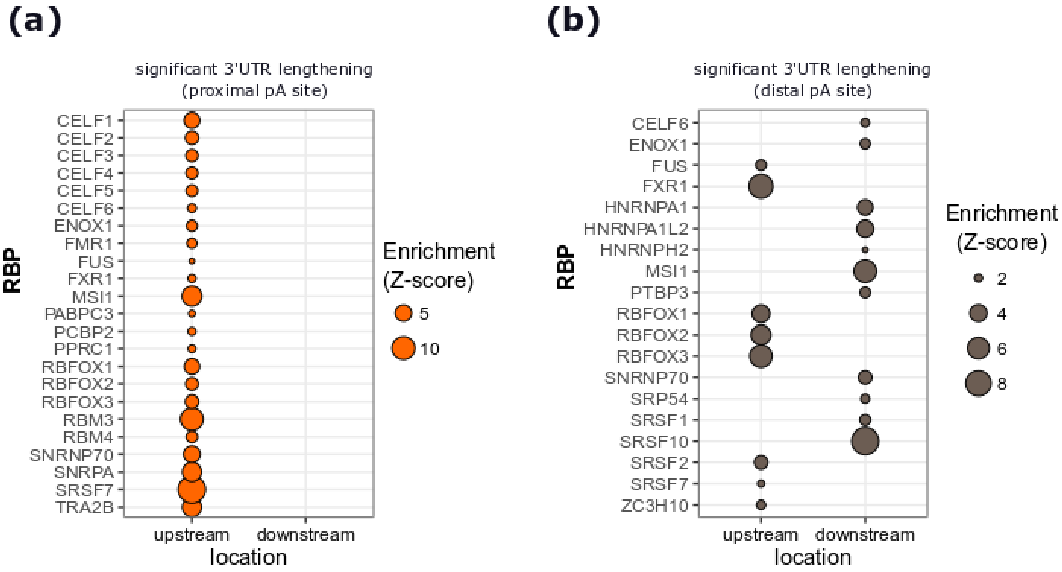

Figure S6: The list of RBPs predicted to have an enrichment of their binding motifs in the 3′UTR lengthening events upon *DSCAM-AS1* silencing. The enrichment is shown for a selected region upstream and downstream of proximal **(a)** and distal **(b)** APA sites, respectively. Significance: z-score > 1.96.

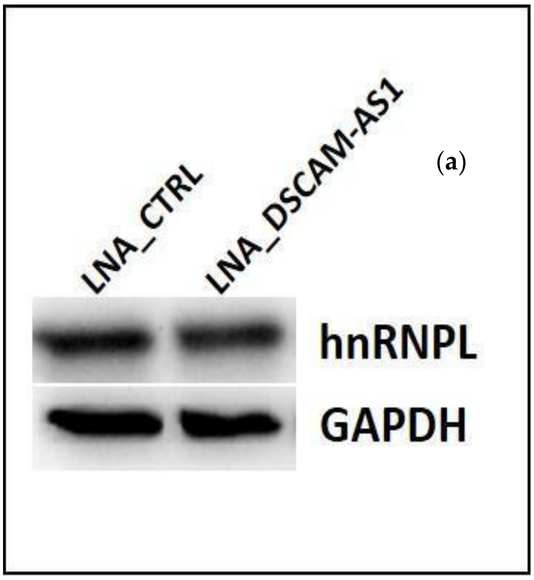

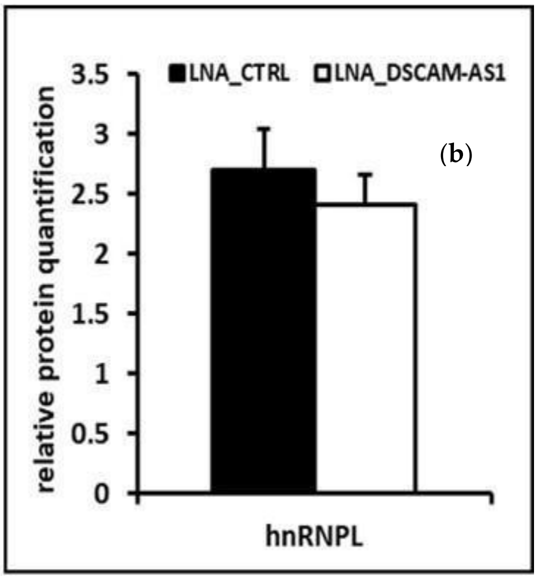

Figure S7. (**a**) Representative image of Western blot analysis of hnRNPL protein, GAPDH: loading control. (**b**) The histogram shows the protein level of hnRNPL in MCF-7 cells transfected with *DSCAM-AS1* or control LNA. hnRNPL values are relative to GAPDH. Error bars represent the standard error of three biological replicates.

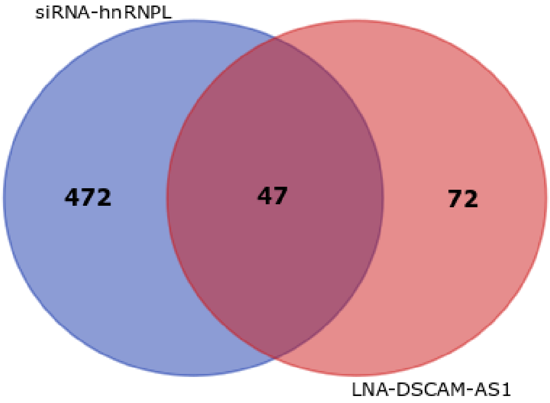

Figure S8. Venn diagram showing the number of overlapping exons significantly regulated in our study and upon siRNA-mediated silencing of hnRNPL in LNCaP prostate cancer cells [15].

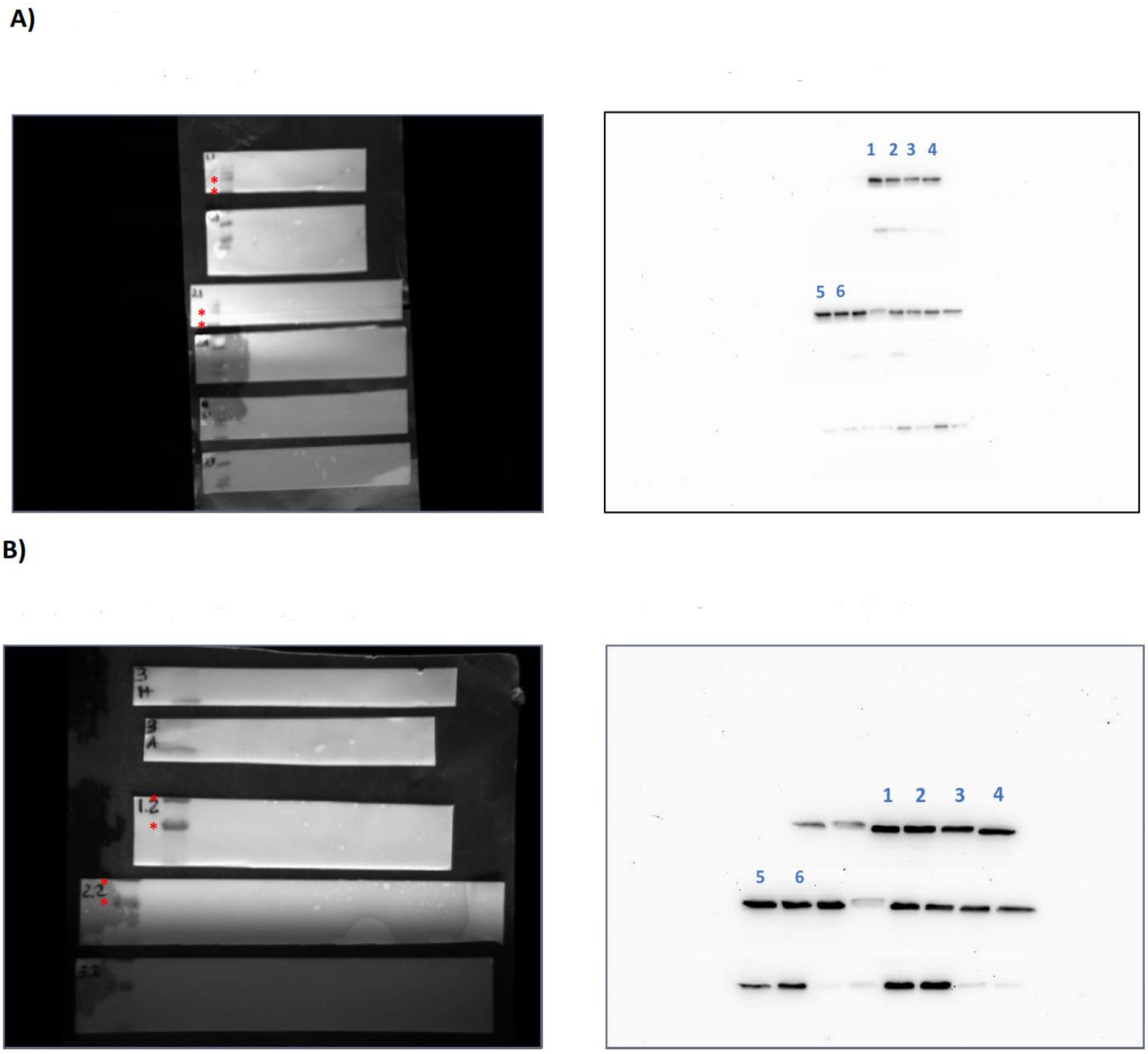

Figure 9. Uncropped western blot image relative to the results showed in Figure S7. (**A**) hnRNPL (Left) Uncropped image of markers. Red stars indicate the bands of the molecular marker at 75 KDa (upper) and 50 KDa (lower). (Right) Uncropped image of hnRNPL. Blue numbers indicate the analyzed bands for hnRNPL protein quantification (predicted MW 68 KDa). (**B**) GAPDH. (Left) Uncropped image of markers. Red stars indicate the bands of the molecular marker at 50 KDa (upper) and 37 KDa (lower). (Right) Uncropped image of GAPDH. Blue numbers indicate the analyzed bands for GAPDH protein quantification (predicted MW 37 KDa). Samples' Legend: 1 = LNA_CTRL replicate 1. 2 = LNA_DSCAM-AS1 replicate 1. 3 = LNA_CTRL replicate 2. 4 = LNA_DSCAM-AS1 replicate 2. 5 = LNA_CTRL replicate3. 6 = LNA_DSCAM-AS1 replicate 3.

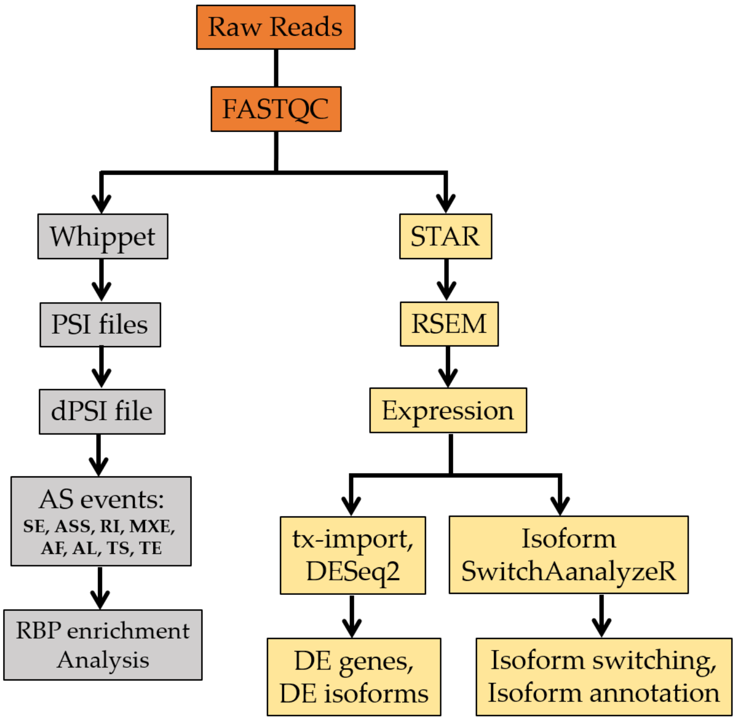

Figure S10. Flow chart showing the different bioinformatic tools and steps used in this study. The FASTQC utility was used for quality check of raw RNA-seq reads. Two pipelines were then applied: (i) shown on the left is the pipeline used for the analysis of alternative splicing changes upon DSCAM-AS1 silencing, using whippet and (ii) on the right is shown the pipeline and tools used for performing differential expression analysis at both gene and isoform levels, as well as isoform switching analysis.

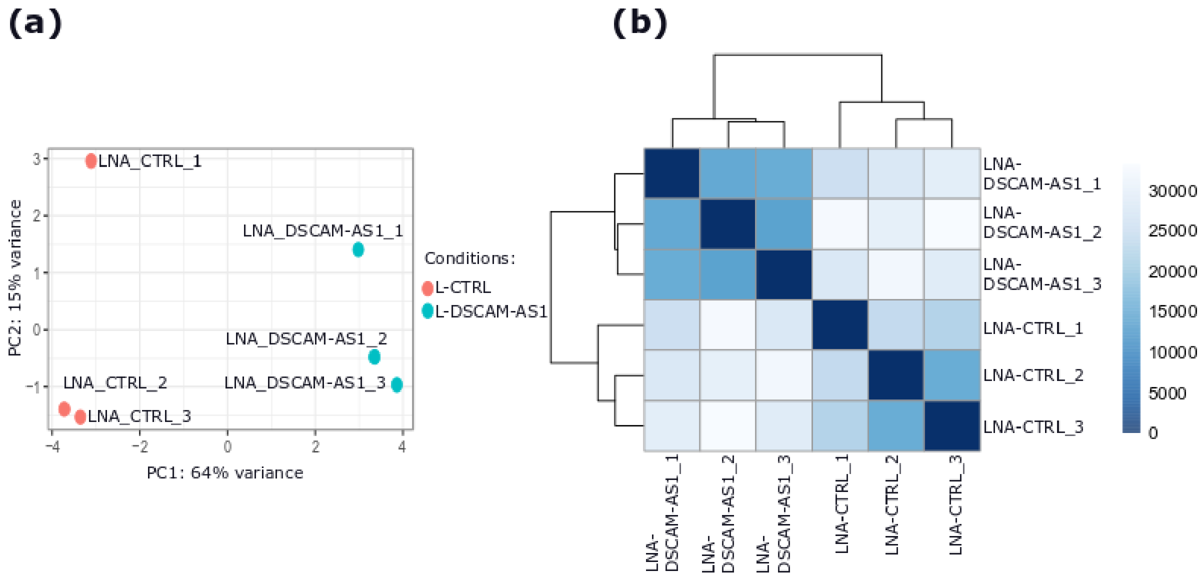

Figure S11. Quality control check of the RNA-seq dataset. **(a)**, a PCA plot showing the separation of replicates based on the gene normalized read counts. **(b)**, heat map reporting the dissimilarity matrix between replicates using the gene normalized read counts.
